# Preliminary Proteomic and Metabolomic Analyses Reveal Potential Serum Biomarkers for Identifying Alveolar Echinococcosis in Mice

**DOI:** 10.3390/vetsci12060565

**Published:** 2025-06-09

**Authors:** Qing Zhang, Xiongying Zhang, Na Liu, Jia Liu, Wei Wang, Yongshun Wang, Wen Lei, Cunzhe Zhao, Wanli Ma, Shuai Guo, Huixia Cai, Jingxiao Zhang, Yufang Liu, Kemei Shi, Wen Zhang, Xiao Ma

**Affiliations:** 1 Qinghai Institute of Endemic Disease Prevention and Control, Xining 811602, China; qhdfbzq@163.com (Q.Z.);; 2 Department of Laboratory Medicine, School of Medicine, Jiangsu University, Zhenjiang 212013, China

**Keywords:** *Echinococcus multilocularis*, proteomics, metabolome, amino acid metabolism, zoonosis

## Abstract

Alveolar echinococcosis (AE) is a chronic and potentially fatal zoonotic parasitic disease that seriously affects the host’s health. It is caused by the proliferation of *Echinococcus multilocularis* larvae within the liver. Due to its long incubation period following host infection, early diagnosis of the disease is currently not feasible. Treatment options are extremely limited, with the only choice being curative surgical resection combined with benzimidazole medication. Thus, the development of early, rapid, and minimally invasive diagnostic methods is crucial for enhancing patient prognosis. This study conducted proteomic and metabolomic analyses of protein and metabolite changes in the serum of a treatment group and control group, aiming to compare the differences between them. Overall, 22 proteins showed significant differences between the treatment and control groups, primarily involved in carbohydrate metabolism, lipid metabolism, and amino acid metabolism. The upregulation of genes related to immune response and enhanced glycolysis were observed, possibly associated with the reproduction of *E. multilocularis* in the liver. A total of 182 metabolites were screened to distinguish between the treatment group and control group. A significant increase in the cytochrome P450 (cP450) metabolite of arachidonic acid indicated signs of renal and splenic involvement in the treatment group. Furthermore, Kyoto Encyclopedia of Genes and Genomes (KEGG) pathway analysis highlighted a strong association between amino acid metabolism and the development of AE. The observed changes in amino acid levels may provide nutrients that facilitate *E. multilocularis* colonization and contribute to the pathogenesis of AE. In summary, by investigating the different characteristics of the AE and control group through proteomic (*n* = 4/group/time point) and metabolomic (*n* = 8/group/time point) analyses, potential serum biomarkers for diagnosing mice with AE were identified.

## Simple Summary

Alveolar echinococcosis (AE), a life-threatening zoonotic disease caused by the larval stage of the tapeworm *Echinococcus multilocularis*, is frequently undetected during early infection and is typically diagnosed at advanced stages through imaging-based methods. Current therapeutic strategies are limited to pharmacological treatment or surgical intervention. To develop improved early diagnostic approaches, in this study, we used eight mice in the infected group and eight mice in the control group to perform metabolomic analysis. In addition, four mice from each group were randomly selected for proteomic analysis based on sample quality and volume. Proteomic and metabolomic analyses identified significant differences in 22 proteins and 182 metabolites between the AE-infected and healthy mice. These alterations suggest potential disruptions in host energy metabolism and multi-organ function during infection. The distinct molecular profiles observed in this study may serve as candidate biomarkers for AE, potentially enabling minimally invasive diagnostic strategies. These findings provide a foundation for the development of rapid, non-invasive early detection methods, which could support timely intervention and improved outcomes, particularly in resource-limited settings where AE is endemic.

## 1. Introduction

Human echinococcosis comprises two severe larval cestode infections: cystic echinococcosis (CE) and alveolar echinococcosis (AE). These conditions are caused by distinct *Echinococcus* species, namely *E. granulosus* sensu lato and *E. multilocularis*, which have divergent transmission patterns and clinical outcomes [1]. CE is predominantly found in regions such as Western China, South America, the Mediterranean, and East Africa [2]. In contrast, AE is primarily observed in the Northern Hemisphere, particularly in Canada [3], France [4], Germany [5], and also in Western China [6]. *E. multilocularis*, a tiny parasite responsible for AE, predominantly triggers hepatic diseases, including liver fibrosis and even the risk of liver cancer, posing a grave threat to human lives [7]. It is estimated that approximately 17,400 new infections occur each year, with the majority of cases happening in China [8]. The intermediate hosts involved in the life cycle of *E. multilocularis* include arvicoline and microtine rodents, as well as small herbivorous mammals. Its definitive hosts include all fox species, wolves, raccoon dogs, domestic dogs, and cats. Humans can become accidental intermediate hosts by ingesting eggs excreted in the feces of definitive hosts, acquiring the infection in the process [1]. In most cases, these eggs hatch into oncospheres in the intestinal tract and migrate through the lymphatic vessels and portal vein to settle in the liver, where they transform into multi-vesicular metacestodes (MCs). MCs exhibit gradual and virtually unrestricted proliferation, resembling malignant tumors. They can infiltrate nearby tissues, leading to a severe chronic AE that becomes lethal without treatment [9,10]. The mortality rate among AE patients who are untreated or inadequately managed reaches approximately 90% within a ten to fifteen years after diagnosis [11]. In rarer instances, they may also reach various organs such as the lungs, brain, bones, or any other organ of the human or intermediate host [1].

The precise molecular mechanism underlying *E. multilocularis*-induced damage to host liver cells during the early and middle stages of infection remains unclear, and no effective medications for reversing liver fibrosis induced by *E. multilocularis* have been successfully developed [12]. Treatment options are limited and primarily consist of radical surgical removal combined with the administration of benzimidazole drugs [13]. Nonetheless, the surgical resection of parasitic tissue is feasible in only a limited fraction of cases (roughly 20%), leaving benzimidazole therapy as the only option for patients ineligible for surgery [14]. Thus, it is crucial to promptly establish early, rapid, minimally invasive, and effective diagnostic methods to enhance the efficacy of liver fibrosis treatment. The liver is the primary organ for various metabolic processes, and its impairment can lead to significant metabolic alterations in the serum. Therefore, metabolic changes in the serum can directly reflect the severity of the disease [15]. Recent studies have provided valuable insights into the exploration of early or follow-up diagnostic biomarkers related to parasitic infections. M. E. Ancarola et al. characterized the profile of extracellular RNAs secreted by in vitro-grown metacestodes of *E. multilocularis*. By analyzing the products secreted into both the external and internal parasite milieus, they demonstrated that the metacestodes exhibit a polarized secretion pattern of various classes of small non-coding RNAs [16]. M. A. Cucher et al. characterized the circulating small RNAs in AE and CE patients, identifying 20 differentially expressed small RNAs associated with AE, CE, and/or non-parasitic lesions [17]. In addition, S. Özdemir et al. identified exosomal circRNAs as potential biomarkers for the detection of hepatic AE [18]. Nonetheless, the specific alterations in metabolic profiles within the serum of individuals afflicted with AE remain enigmatic. Unraveling these metabolic fluctuations holds the potential to enhance our comprehension of AE’s disease mechanisms and furnish novel, pertinent biomarkers for clinical assessment. Serum proteomics and metabolomics, respectively, provide information about proteins and small-molecule metabolites within the biological system. Joint analysis contributes to a more comprehensive understanding of biological processes and disease development within the organism, thus aiding in the discovery of novel biomarkers for early diagnosis.

This research project is based on the assumption that AE triggers distinct molecular alterations within the serum of mice, which are discernible. To substantiate this conjecture, we utilized proteomic and metabolomic methodologies to scrutinize the serum proteome and metabolome [19] of AE-infected mice.

## 2. Materials and Methods

### 2.1. Establishment of AE Mouse Model

These experiments utilized a secondary infection method by intraperitoneally injecting protoscoleces into experimental mice to induce infection with *E. multilocularis*, thereby establishing the AE mouse model. The protoscoleces were obtained through breeding gerbils at the Plateau Medical Research Center. After humane euthanasia via cervical dislocation, the cysts were dissected and their contents were placed in a flat dish with physiological saline. They were then finely minced and filtered through an 80-mesh sieve to obtain a filtrate. The filtrate was subsequently washed multiple times with physiological saline to obtain a clear solution containing protoscoleces. After thoroughly mixing the solution, 50 μL was drawn onto a glass slide, followed by the quick addition of a drop of 0.03% methylene blue staining solution. A coverslip was placed over the slide for microscopic examination. The total number of protoscoleces was counted, and the survival rate was calculated (assessment criteria: protoscoleces appearing transparent were considered alive, while those stained blue by methylene blue were considered dead). In this experiment, the survival rate was found to be 90.96% (302/332).

The protoscoleces solution was reconstituted to a final concentration of 8–9 protoscoleces per microliter using penicillin–streptomycin-supplemented saline (800 units of streptomycin and 1000 units of penicillin per milliliter). Subsequently, mice in the experimental group (*n* = 32) were intraperitoneally injected with 0.3 mL of the aforementioned mixture (approximately 2500 protoscoleces), while the control group (*n* = 32) mice received an intraperitoneal injection of 0.3 mL of double-antibiotic physiological saline each. The mice were then raised and observed. All the mice were housed in isolated ventilated cages in a barrier facility at the Qinghai Institute of Endemic Disease Prevention and Control Laboratory Animal Center. The mice were maintained on a 12/12 h light/dark cycle at 20–26 °C with sterile pellet food and water ad libitum.

### 2.2. Sample Collection and Processing

At 45, 90, 135, and 180 days post-inoculation, blood samples were collected from 4 mice in the experimental group and 4 mice in the control group. These mice were randomly selected from a larger cohort of 32 mice per group. For metabolome analysis, blood samples were collected from eight mice in each group for further research (Supplementary Materials Figure S1). All the mice used in proteomic and metabolomic analysis were selected based on sample integrity and volume adequacy. The blood was collected and stored in a clean 2 mL Eppendorf tubes. The collected blood volume was recorded. This process was conducted out of sight of the other mice. Subsequently, the mice were euthanized by cervical dislocation, and the number, size, and location of lesions were documented. In comparison to the control group mice, the experimental group mice exhibited milky-white cysts on the liver surface. As the duration of infection increased, these cysts on the liver capsule surface enlarged, and the lesions gradually spread into the surrounding liver tissue, significantly disrupting the normal structure of the liver tissue. The collected blood was kept at 4 °C for 3 h. Subsequently, the blood was centrifuged at 3000 rpm and 4 °C for 10 min to separate the serum, which was then transferred to new Eppendorf tubes and stored at −80 °C for later use.

The protein extraction was performed using the SDT method (4% (*w*/*v*) SDS, 100 mM Tris/HCl pH 7.6, 0.1M DTT) [20]. Protein quantification was carried out using the Bicinchoninic acid (BCA) assay [21,22]. An appropriate amount of protein from each sample was subjected to enzymatic digestion using the Filter-Aided Proteome Preparation (FASP) method. Peptide desalting was carried out using a C18 Cartridge, followed by the freeze-drying of the peptides. The dried peptides were reconstituted in 40 μL of 0.1% formic acid solution, and peptide quantification was performed using the OD280 method. Each sample was taken at 100 μg of peptide and labeled according to the manufacturer’s instructions for the TMT reagents [23]. The labeled peptides from each group were mixed in equal amounts and fractionated using a High-pH Reversed-Phase Peptide Fractionation Kit. Initially, column equilibration was performed using acetonitrile and 0.1% trifluoroacetic acid (TFA). Subsequently, the mixed labeled peptide samples were loaded onto the column, followed by desalting through low-speed centrifugation after adding purified water. Finally, a gradient elution was carried out using a high-pH acetonitrile solution with increasing concentrations to elute the column-bound peptide. After each elution, the peptide samples were vacuum-dried and reconstituted in 12 μL of 0.1% FA, followed by OD280 measurement to determine peptide concentrations.

### 2.3. LC-MS/MS Analysis

Each sample was separated using the nanoflow High-Performance Liquid Chromatography (HPLC) system Easy nLC. Buffer A was a 0.1% formic acid aqueous solution, and Buffer B was a 0.1% formic acid in acetonitrile solution (acetonitrile content 84%). The chromatographic column was equilibrated with 95% Buffer A, and the samples were automatically loaded onto a loading column (Thermo Scientific Acclaim PepMap100, 100 μm × 2 cm, nanoViper C18), followed by separation on an analytical column (Thermo Scientific EASY column, Waltham, MA, USA, 10 cm, ID 75 μm, 3 μm, C18-A2) at a flow rate of 300 nL/min. After chromatographic separation, the samples were subjected to mass spectrometry analysis using a Q-Exactive mass spectrometer. The detection mode was positive ion, with a parent ion scan range of 300–1800 *m*/*z*. The first-level mass resolution was set at 70,000 at 200 *m*/*z*, the AGC (Automatic Gain Control) target was 1 × 10^6^, the Maximum IT (Maximum Ion Time) was 50 ms, and the dynamic exclusion time was 60.0 s. Peptide and peptide fragment mass-to-charge ratios were acquired using the following parameters: after each full scan, 20 fragmentation spectra (MS2 scans) were collected. The MS2 Activation Type was set to HCD, with an isolation window of 2 *m*/*z*. The second-level mass resolution was 17,500 at 200 *m*/*z*, the Normalized Collision Energy was 30 eV, and the Underfill was set at 0.1%.

### 2.4. Mass Spectrometry Data Analysis and Protein Quantification

The retrieval software used for this study was Proteome Discoverer v1.4 [24], with Mascot v2.2 integrated. The search parameters were as follows: The database employed was the Swissprot_mouse_17097_20220104.fasta database, consisting of a total of 17,097 sequences. To control the FDR resulting from random matches, a decoy database (Reverse) was utilized. The quantification method was set to TMT. The fixed modifications were Carbamidomethyl (C), TMT6/10/16 plex (N-term), and TMT6/10/16 plex (K), and the variable modifications were Oxidation (M) and TMT6/10/16 plex (Y). Trypsin was used as the digestion enzyme, with a maximum of 2 missed cleavages allowed. The peptide mass tolerance was set at 20 ppm, and the fragment mass tolerance was set at 0.1 Da. The data were filtered at a peptide-level FDR of 1% to retain reliable protein identifications. To ensure high-quality analysis results, each protein was required to contain at least one unique peptide in the search results. The filtered data were subsequently used for downstream analysis. Protein quantification in Proteome Discoverer using Isobaric labeling was accomplished by summing the intensities of the unique peptides. After completing the protein quantification, it was necessary to extract the intensity values for each protein from the search results for different samples. Subsequently, these values underwent centralization transformation to perform sample-wise normalization, resulting in relative protein quantification values across the different samples.

### 2.5. Procedure for Metabolomic Sample Extraction and Data Preprocessing

Each blood sample was vortexed for 10 s to ensure thorough mixing, following which 50 μL of the sample was transferred into the corresponding labeled centrifuge tube. Subsequently, 300 μL of a 20% acetonitrile–methanol internal standard extraction solution was added to each tube, and the mixtures were vortexed for 3 min. The samples were then centrifuged at 12,000 rpm for 10 min at 4 °C. After centrifugation, 200 μL of the supernatant was transferred to a new centrifuge tube and incubated at −20 °C for 30 min. Finally, the samples underwent an additional 3 min centrifugation at 12,000 rpm, and the resultant supernatant (180 μL) was transferred into the corresponding sample vial insert for subsequent analysis [25]. The raw data were converted to mzXML format using ProteoWizard [26], and peak extraction, alignment, and retention time correction were performed using the XCMS program. Peak areas were corrected using the “SVR” method, and peaks with a missing rate > 50% in each sample group were filtered out.

### 2.6. Statistical Analysis

Statistical analyses and data normalization were performed using custom Python (v3.11) scripts and R software (v4.0.3). Visualization was conducted with the R packages ggplot2, ggpubr, ComplexHeatmap, and igraph. PCA was performed using the prcomp function with unit variance scaling. Venn diagrams were generated using VennDiagram, and subcellular localization was predicted using WoLF PSORT. GO and pathway enrichment analyses were carried out using clusterProfiler. Significant differences were defined as *p* < 0.05 with VIP > 1 or fold change >1.5.

### 2.7. Data Availability

The mass spectrometry proteomics data were deposited in the ProteomeXchange Consortium (http://proteomecentral.proteomexchange.org (accessed on 6 October 2023)) via the iProX partner repository [27,28] with the dataset identifier PXD045937. The raw metabolomics data generated and analyzed in this study are publicly available in the MetaboLights repository under the accession number MTBLS12532.

## 3. Results

### 3.1. Differential Serum Protein Profiling and Functional Enrichment Reveal Potential Biomarkers and Key Biological Pathways in Treatment Group

**Figure 1:**
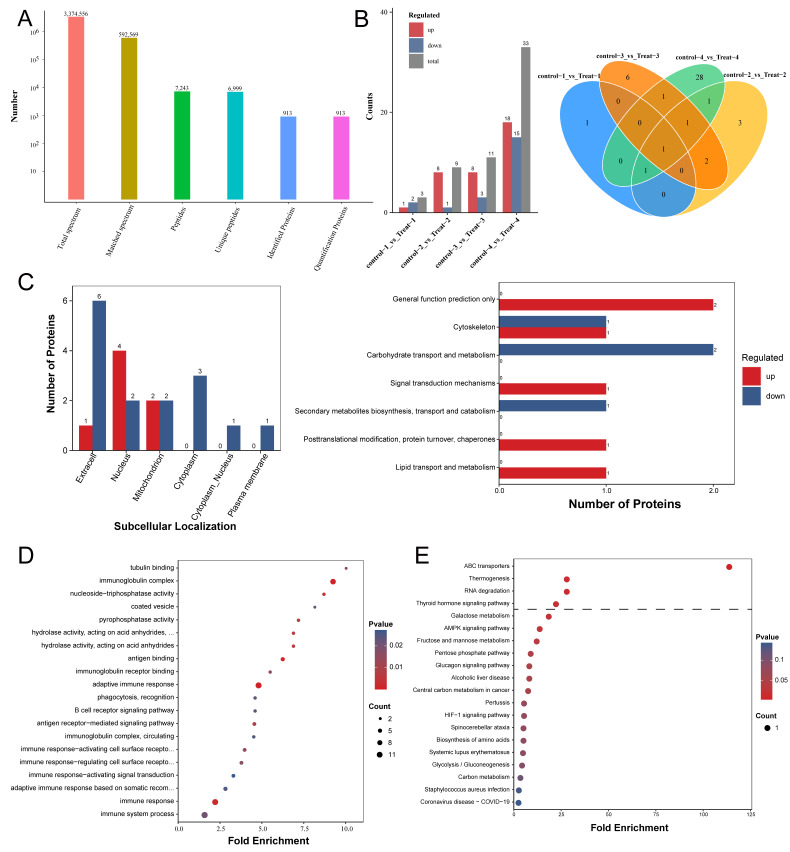
The functional enrichment analysis of the differentially abundant proteins in AE mouse serum. (**A**) The protein level identification results. (**B**) On the left are bar charts showing the differential proteins detected between the control and treated groups at the four sampling time points; on the right are Venn diagrams displaying the shared and unique proteins between the control and treated groups at each of the four sampling time points. (**C**) On the left is a bar chart comparing the upregulated and downregulated subcellular localization results. The horizontal axis represents subcellular compartments; the vertical axis represents the number of differentially expressed proteins annotated to each subcellular compartment, with red and blue colors indicating the upregulated and downregulated differentially expressed proteins, respectively. On the right is a bar chart comparing the upregulated and downregulated KOG annotations. The horizontal axis represents the number of differentially expressed proteins annotated to each functional category, while the vertical axis displays the KOG functional classification names, with red and blue colors indicating the upregulated and downregulated differentially expressed proteins, respectively. (**D**) A bubble chart depicting protein enrichment and differential abundance across molecular functions, biological processes, and cellular components. (**E**) A bubble diagram of the enrichment of the differentially abundant proteins in the Kyoto Encyclopedia of Genes and Genomes (KEGG).

In the LC-MS/MS analysis, a total of 3,374,556 spectra were detected, of which 592,569 were deemed effective and usable. Employing a peptide-level FDR of less than 1%, we successfully identified 6999 unique peptides among the 7243 initially detected. Moreover, all 913 proteins were quantifiable in the analysis (Figure 1A). We analyzed the differences in serum protein expression between the control and treated groups of mice at four different sampling time points. As the infection progressed, the number of differential proteins between the two groups gradually increased, ranging from 3 to 33 (Figure 1B). Notably, the expression level of Chitinase-like protein 3 (CHIL3; UniProt accession number: O35744) was significantly higher in the treated group compared to the control group at all four sampling time points. CHIL3 proteins, produced by various cell types, are most notably recognized as biomarkers secreted by M2 macrophages in a range of diseases [29,30]. Moreover, some studies suggest that CHIL3 has beneficial effects on regulating hepatocellular metabolism and mitochondrial activities following hepatic ischemia–reperfusion. The addition of CHIL3 alone was shown to improve hepatocellular metabolism and reduce damage caused by ischemic or oxygen–glucose deprivation in cultured mouse and human hepatocytes [31]. Additionally, the levels of Complement C4-B (P01029) and Ig kappa chain V-II region 26-10 (P01631) proteins were also significantly elevated at three of the four sampling time points in the treatment group compared to the control group (Figure 1B and Supplementary Materials Table S1). Overall, 22 proteins exhibited a significant difference (FC > 1.5; *p* < 0.05) between the two groups, with 7 proteins upregulated and 15 proteins downregulated (Supplementary Materials Table S1 and Figure S2). In order to determine the characteristics of the proteins exhibiting differential abundance, we performed subcellular localization and KOG annotation for this set of 22 proteins. The subcellular localization annotation indicated that, out of the 22 differentially abundant proteins, 7 were localized in the extracellular space, 6 were localized in the nucleus, 4 were localized in the mitochondrion, 3 were localized in the cytoplasm, 1 was localized in the cytoplasm and nucleus, and 1 was localized in the plasma membrane (Figure 1C and Supplementary Materials Table S2). The differential proteins with diverse functions in the KOG Functional classification played roles in the cytoskeleton; carbohydrate transport and metabolism; secondary metabolite biosynthesis, transport, and catabolism; lipid transport and metabolism; and signal transduction mechanisms, as well as posttranslational modification, protein turnover, and chaperones (Figure 1C).

Bioinformatic analysis was conducted to discern the principal biological pathways and functional classifications within the set of differentially abundant proteins (FC > 1.5; *p* < 0.05). The classification of proteins using GO revealed differences between the two groups of mice in biological processes such as immune response, adaptive immune response, and immune system processes. Variations were also observed in cellular components like the immunoglobulin complex and circulating proteins. Furthermore, distinctions existed in molecular functions such as antigen binding and immunoglobulin receptor binding (Figure 1D and Supplementary Materials Table S3). KEGG analysis revealed that the most significant altered pathways involved ABC transporters, RNA degradation, thermogenesis, and the thyroid hormone signaling pathway (Figure 1E and Supplementary Materials Table S4). In general, proteomic analysis revealed that the biological pathways involving serum proteins in mice infected with *E. multilocularis* differed from those in normal mice.

### 3.2. Untargeted Metabolomics Detected Changes in Mice Serum Metabolites

**Figure 2:**
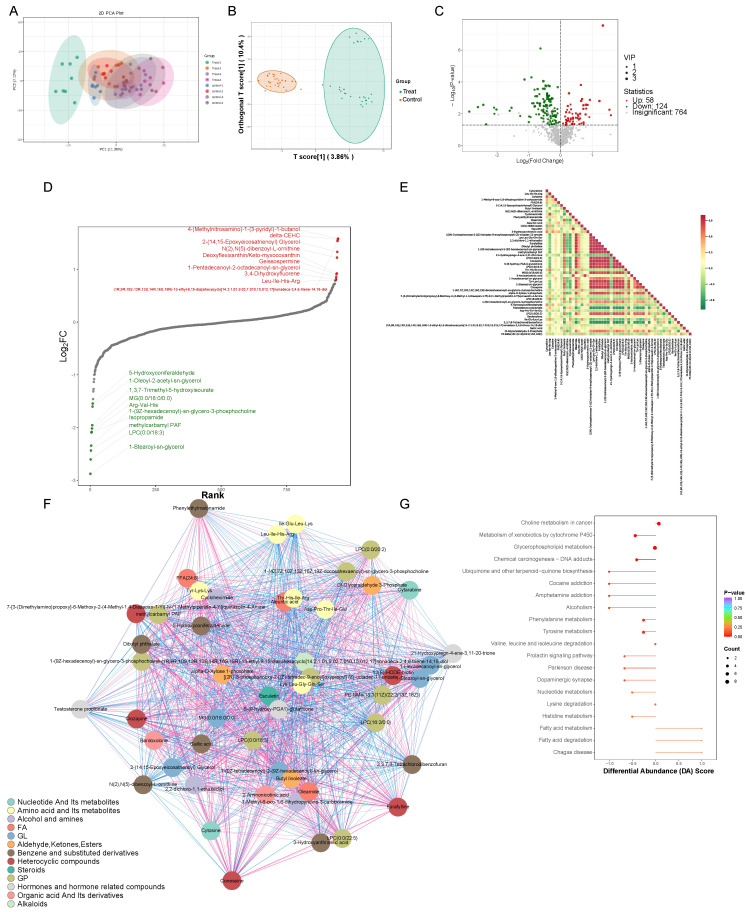
The metabolomic analysis of serum samples from the treatment group. (**A**) A Principal Component Analysis plot (PCA plot) where the horizontal axis represents PC1 and the vertical axis represents treatment group PC2, representing the scores of the first and second principal components, respectively. The differently colored dots represent samples from different groups. (**B**) A Partial Least Squares Discriminant Analysis score plot (OPLS-DA Score plot), where the horizontal axis represents the predictive component scores, showing differences between groups in the horizontal direction; the vertical axis represents the orthogonal component scores, indicating differences within groups in the vertical direction; the percentages denote the explanatory power of the components on the dataset. (**C**) A volcano plot of the differential metabolites, where each point in the volcano plot represents a metabolite. The green points represent downregulated differential metabolites, red points represent upregulated differential metabolites, and gray points represent metabolites that were detected but were not significantly different. The horizontal axis represents the logarithmic fold change (log2FC) in the relative abundance of a metabolite between the two sample groups, with larger absolute values indicating greater differences in relative abundance between the groups. Under the selection criteria of VIP + FC + *p*-value, the vertical axis represents the level of significance (-log10*p*-value) and the size of the circles represents the VIP value. (**D**) A dynamic distribution chart of differential metabolite contents, where the horizontal axis represents the cumulative number of substances sorted by fold change from small to large, and the vertical axis represents the logarithmic values of the fold changes with a base of 2. Each point represents a substance, with the green points indicating the top 10 most downregulated substances and red points indicating the top 10 most upregulated substances. (**E**) A differential metabolite correlation heatmap where both the horizontal and vertical axes feature the names of differentially abundant metabolites. The different colors indicate the strength of the Pearson correlation coefficients: red represents stronger positive correlation, green represents stronger negative correlation, and darker colors indicate larger absolute correlation coefficients between samples. (**F**) A differential metabolite correlation network graph where the nodes represent significantly different metabolites and node size corresponds to their degree of connectivity, with larger nodes having more connections. The pink lines represent positive correlations, while the blue lines represent negative correlations. The thicknesses of the line reflects the absolute magnitude of the correlation coefficients, with thicker lines indicating stronger correlations. (**G**) A differential abundance score plot where the vertical axis represents differential pathway names (sorted by *p*-value) and the horizontal axis represents differential abundance scores (DA Scores). The DA Scores reflect the overall change in expression of all the metabolites in a metabolic pathway. A score of 1 indicates an overall upregulation of all the identified metabolites in that pathway, while a score of −1 indicates an overall downregulation of all the identified metabolites. Larger circles represent greater numbers of metabolites. The colors of the line segments and circles reflect the magnitude of the *p*-value, with a closer proximity to red indicating smaller *p*-values and a closer proximity to purple indicating larger *p*-values.

By performing Principal Component Analysis (PCA) on the samples, our objective was to acquire preliminary insights into the general metabolic distinctions among sample groups and the extent of variability within each group. The outcomes illustrated that the mouse serum metabolomics dataset exhibited good stability and reproducibility (PERMANOVA, *p* < 0.001) (Figure 2A and Supplementary Materials Table S5). A total of 1348 metabolites were detected, categorized into 22 classes, including Organic Acids and Their Derivatives, Carbohydrates and Their Metabolites, Aldehydes, Ketones, Esters, Nucleotides and Their Metabolites, Benzene and Substituted Derivatives, and Amino Acids and Their Metabolites, among others (Supplementary Materials Figure S3 and Table S5). In order to investigate the detailed variables more comprehensively, we performed an OPLS-DA analysis on all the metabolic profiles. As illustrated in Figure 2B within the score plots of the OPLS-DA models, significant distinctions in data clustering were clearly observable between the experimental group and the control group. A volcano plot was used to display the relative abundance differences in the metabolites between the two groups of samples and their statistical significance. The metabolic alterations were assessed using a threshold of Variable Importance in Projection (VIP) > 1 and a significance level of *p* < 0.05, with the metabolites exhibiting significant changes depicted using green or red dots and larger circular shapes. The results showed 58 upregulated metabolites and 124 downregulated metabolites (Figure 2C and Supplementary Materials Table S6). To gain a clearer understanding of the overall metabolic differences, a dynamic distribution plot of metabolite content disparities was created. The top 10 upregulated and downregulated metabolites were annotated. The results revealed that the most significantly upregulated metabolite belonged to the Heterocyclic compounds category, namely, 4-(Methylnitrosamino)-1-(3-pyridyl)-1-butanol (NNK), while the most pronounced downregulation was observed in the Glycerolipids (GLs) category with 1-Stearoyl-sn-glycerol (Figure 2D). In the serum of both groups, significant correlations were observed among metabolites such as amino acids, nucleotides, organic acids, and steroids. This elucidated a complex network of interactions, indicating that echinococcosis had a broad impact on serum metabolism (Figure 2E,F). In addition, significant changes in the following metabolic pathways were observed in the treatment group of mice: glycerophospholipid metabolism, the metabolism of xenobiotics by cytochrome P450, choline metabolism in cancer, tyrosine metabolism, and phenylalanine metabolism (Figure 2G).

### 3.3. Cross-Analysis of Proteomics and Metabolomics Highlights Altered Amino Acid Metabolism in AE-Infected Mice

**Figure 3:**
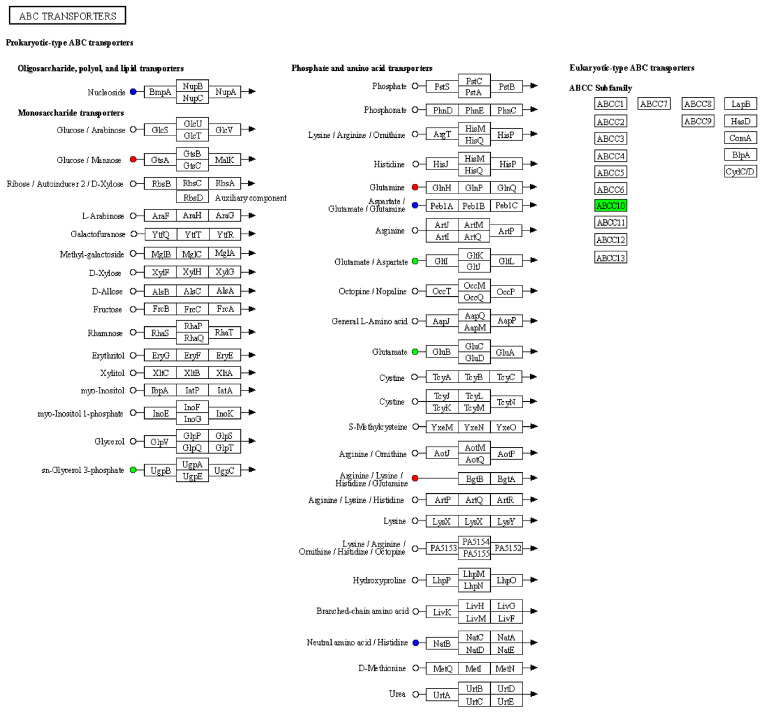
Integration of proteomics and metabolomics data. Kyoto Encyclopedia of Genes and Genomes (KEGG) pathway diagram, where nodes and squares represent metabolites and proteins, respectively. Red indicates upregulated proteins/metabolites, green indicates downregulated proteins/metabolites, and blue represents pathways containing both upregulated and downregulated protein.

Based on the results of the differential metabolite and protein analyses, we simultaneously mapped differential proteins and metabolites from the same comparison group onto KEGG pathway maps to gain a better understanding of the relationship between proteins and metabolites. The results revealed some proteins with abnormal expression in the ‘ABC transporters’ pathway, including the upregulation of arginine, lysine, histidine, and glutamine expression, as well as downregulation of glutamate and aspartate expression (Figure 3). The above results indicated that serum amino acid metabolism pathways were abnormal in mice infected with AE. Based on this, it can be inferred that amino acids may be the main nutritional substances consumed by *E. multilocularis*, consequently resulting in nutritional loss in mice.

## 4. Discussion

AE is a serious zoonotic disease that poses a significant threat to both human and animal health. Globally, approximately 18,235 new cases are reported each year [32]. Due to the lack of effective early diagnostic tools and treatment options, patients often suffer from severe physical distress and substantial economic burdens. In comparison to tissue biopsy, liquid biopsy provides multiple advantages, such as minimally invasive sampling, efficient disease monitoring, and the ability to perform longitudinal evaluations of therapeutic responses [33,34]. In this study, we successfully established an AE mouse model and observed characteristic changes in the mice’s liver tissue, laying the foundation for subsequent experimental analysis. Compared to single-omics analysis, integrated multi-omics analysis [35] offers a more comprehensive insight into the biological mechanisms and molecular features underlying murine *E. multilocularis* infection. We conducted proteomic and metabolomic analyses on murine sera at various stages of *E. multilocularis* infection, resulting in a substantial amount of interpretable data. We selected a total of 22 proteins to distinguish between the treatment group and control group. The differential proteins revealed their involvement in carbohydrate metabolism, lipid metabolism, and amino acid metabolism, reflecting the characteristics of AE occurrence and progression. We observed the upregulation of genes involved in immune response, such as C4b and Dennd1b [36], in the serum of the treatment group mice. This is likely directly related to the infection by *E. multilocularis*. Additionally, the aberrant expression of the Pfkl gene may have adverse effects on glucose metabolism, energy balance, and potentially be associated with tumorigenesis [37]. This result was also validated in the GO analysis. Through KEGG analysis, we determined that the molecular characteristics of the AE mouse model were primarily associated with amino acid metabolism, glycolysis/gluconeogenesis, and other biological processes. The suppression of glycolysis and gluconeogenesis pathways can serve as an indicator of metabolic and energy imbalance within the organism, which may be related to the colonization and reproduction of *E. multilocularis* in the liver [38]. There are reports indicating that *E. multilocularis* protoscoleces enhance glycolysis to promote immune responses, which aligns with the findings of this study [7,39]. The differential proteins in the sera are closely associated with *E. multilocularis* infection and can serve as potential target proteins for studying the pathogenic mechanisms of AE in mice.

In this study, we analyzed and elucidated changes in the mouse serum metabolome induced by AE. We screened a total of 182 metabolites to differentiate between the treatment group and control group. In the serum of the treatment group of mice, representative substances like NNK, known for its tumor-inducing potential in experimental animals [40], exhibited a substantial increase in levels. Furthermore, the cP450 metabolite of arachidonic acid, 2-(14,15-Epoxyeicosatrienoyl) Glycerol [41], primarily synthesized in the kidney, spleen, and brain, displayed a notable elevation. This not only indicated damage to liver parenchymal cells in the treatment group but also revealed signs of involvement in organs such as the kidneys and spleen. The observed lesions in the spleens of the experimental mice also corroborate this inference (Supplementary Materials Figure S1). Furthermore, the significant downregulation of lipids such as 1-Stearoyl-sn-glycerol, LPC (0:0/18:3), and methylcarbamyl PAF [42] suggests a negative regulatory effect of *E. multilocularis* infection on cell signaling, energy storage, and platelet-activating factors.

The primary affected organ in AE is the liver (Supplementary Materials Figure S1), and as the duration of parasitic infection increases, *E. multilocularis* can induce structural and functional changes in the liver [11,43]. Through KEGG pathway analysis, we have identified abnormalities in the serum proteins and metabolites of the treatment group of mice (Figure 3), with the majority of them associated with abnormalities in amino acid metabolism, indicating a close correlation between amino acid metabolism and the occurrence and progression of echinococcosis. The catabolism of amino acids primarily occurs in the liver [44]. Consequently, liver damage can lead to alterations in amino acid metabolism, characterized by a decrease in free branched-chain amino acids and an increase in free amino acids (glutamine, lysine, histidine) [45,46]. These variances could offer fresh perspectives on the biological mechanisms implicated in AE infection. Furthermore, in a recent report, a significant decrease in serum aspartate was observed in non-active AE patients suffering from severe muscle atrophy and weight loss, which aligns with the findings of this study [47]. We also observed a significant decrease in serum glutamate levels, a precursor to glutamine [48,49], in the treatment group of mice. This decrease could be attributed to the excessive energy consumption caused by the overgrowth of *E. multilocularis* in liver tissues, surpassing the energy provided by glucose in the body. As a result, glutamine may be utilized as an alternative source, leading to a sharp decline in glutamate levels. However, in some studies, a significant decrease in serum branched-chain amino acids and aromatic amino acids has been observed in AE patients [15,50]. Collectively, our results suggest that amino acid metabolism is altered in infected mice and may influence host–parasite metabolic interactions [46,51]. The changes in the levels of these amino acids may also potentially provide nutrients for *E. multilocularis* colonization within the host and contribute to the pathogenesis of AE, such as the development of liver fibrosis [52]. This study also has some limitations. Firstly, the relatively small sample size of collected mouse samples warrants further validation in a larger and more diverse cohort. Secondly, there is a disparity in sample size between the proteomic and metabolomic analyses, as metabolomics analysis typically requires a relatively larger number of samples to support the reliability and reproducibility of the study. Lastly, specific serum biomarkers have not been definitively identified, and combining transcriptomics or other methods may offer better prospects.

## 5. Conclusions

This study integrated proteomic and metabolomic approaches to characterize protein and metabolic alterations in mice with AE, providing new insights into disease-associated changes and potential diagnostic markers. A total of 22 differentially expressed proteins were identified in AE-infected mice, predominantly associated with carbohydrate metabolism, lipid metabolism, and amino acid metabolism. In total, 182 differential metabolites distinguished the treatment group from the control group, with significant perturbations in glycerophospholipid metabolism, cytochrome P450-mediated xenobiotic detoxification, and amino acid metabolism. Cross-omics analysis highlighted amino acid metabolism as a key node altered by E. multilocularis infection, likely due to parasite nutrient scavenging and host metabolic reprogramming. Dysregulated pathways (e.g., ABC transporters, glycolysis/gluconeogenesis) indicated disrupted hepatic metabolic homeostasis. The identified serum proteins (e.g., CHIL3) and metabolites (e.g., Glycerol and glutamate depletion) may serve as potential indicators of AE-associated metabolic changes. However, due to the lack of validation experiments, their biomarker roles remain preliminary and require further investigation. Despite the limitations of sample size and methodological differences, this study provides a basis for the further exploration of AE pathogenesis and offers clues for future diagnostic strategies.

## Data Availability

The mass spectrometry proteomics data have been deposited in the ProteomeXchange Consortium (http://proteomecentral.proteomexchange.org (accessed on 6 October 2023)) via the iProX partner repository with the dataset identifier PXD045937.

## Supplementary Materials

The following supporting information can be downloaded at: https://www.mdpi.com/article/10.3390/vetsci12060565/s1, Figure S1: Sampling records for two mouse cohorts; Figure S2: Volcano plots of differentially expressed proteins; Figure S3: Metabolomie supporting data for AE, mouse serum samples; Table S1: Differential protein information; Table S2: Differential protein subcellular localization analysis information; Table S3: Differential protein GO enrichment analysis information; Table S4: KEGG analysis information; Table S5: Detailed information on identified mouse serum metabolites; Table S6: Detailed information on differential metabolites.
