# Supplementary material for: Preliminary Proteomic and Metabolomic Analyses Reveal Potential Serum Biomarkers for Identifying Alveolar Echinococcosis in Mice
Source: Vet Sci. 2025 Jun 9;12(6):565. doi: 10.3390/vetsci12060565 (PMC12197404; doi:10.3390/vetsci12060565)
Supplement: Supplementary file 1 [file vetsci-12-00565-s001.zip › Materials Figure S1.pdf]

**Materials Figure S1** Sampling records for two mouse cohorts

| Sample number | period of inoculation | Blood volume (μL) | Serum collection volume (μL) | Pathological lesion | Lesion site | Lesion size (mm) | Multi-omics analysis |
|---|---|---|---|---|---|---|---|
| **1-1** | 45 | 1200 | 400 | Y | Liver | 2*1.5 | Y |
| **1-2** | 45 | 1300 | 400 | Y | Liver | 1*1 | Y |
| **1-3** | 45 | 1100 | 350 | Y | Liver | 1*1 | Y |
| **1-4** | 45 | 1400 | 420 | Y | Liver | 1*2 | Y |
| **1-5** | 45 | 1200 | 400 | Y | Liver | 1*1.5 | N |
| **1-6** | 45 | 1200 | 400 | Y | Liver | 1*1 | N |
| **1-7** | 45 | 1200 | 380 | Y | Liver | 1*1 | N |
| **1-8** | 45 | 1200 | 400 | Y | Liver | 1*1.5 | N |
| **1-1 (-)** | 45 | 1200 | 350 | N | - | - | Y |
| **1-2 (-)** | 45 | 1300 | 380 | N | - | - | Y |
| **1-3 (-)** | 45 | 1100 | 300 | N | - | - | Y |
| **1-4 (-)** | 45 | 800 | 250 | N | - | - | Y |
| **1-5 (-)** | 45 | 1000 | 300 | N | - | - | N |
| **1-6 (-)** | 45 | 1200 | 350 | N | - | - | N |
| **1-7 (-)** | 45 | 1000 | 300 | N | - | - | N |
| **1-8 (-)** | 45 | 1100 | 320 | N | - | - | N |
| **2-1** | 90 | 1150 | 350 | Y | Liver | 8*10 | Y |
| **2-2** | 90 | 1550 | 400 | Y | Liver | 10*10 | Y |
| **2-3** | 90 | 1450 | 400 | Y | Liver | 3*5 | Y |
| **2-4** | 90 | 800 | 350 | Y | Liver | 2*5 | Y |
| **2-5** | 90 | 750 | 360 | Y | Liver | 3*3 | N |
| **2-6** | 90 | 1500 | 550 | Y | Liver, chest diaphragm | 9*14 | N |
| **2-7** | 90 | 1050 | 400 | Y | Liver, mesentery | 10*13 | N |
| **2-8** | 90 | 1250 | 430 | Y | Liver | 4*5 | N |
| **2-1 (-)** | 90 | 1400 | 500 | N | - | - | Y |
| **2-2 (-)** | 90 | 800 | 330 | N | - | - | Y |
| **2-3 (-)** | 90 | 1200 | 400 | N | - | - | Y |
| **2-4 (-)** | 90 | 1200 | 380 | N | - | - | Y |
| **2-5 (-)** | 90 | 1250 | 380 | N | - | - | N |
| **2-6 (-)** | 90 | 1200 | 400 | N | - | - | N |
| **2-7 (-)** | 90 | 1300 | 500 | N | - | - | N |
| **2-8 (-)** | 90 | 1200 | 450 | N | - | - | N |
| **3-1** | 135 | 1200 | 380 | Y | Liver, abdominal cavity, spleen | 7*10 | Y |
| **3-2** | 135 | 1300 | 420 | Y | Liver, abdominal cavity | 3*5 | Y |
| **3-3** | 135 | 1200 | 350 | Y | Liver, abdominal cavity | 10*14 | Y |
| **3-4** | 135 | 1400 | 480 | Y | Liver, abdominal cavity | 12*16 | Y |
| **3-5** | 135 | 1400 | 450 | Y | Liver, abdominal cavity | 5*5 | N |
| **3-6** | 135 | 1500 | 600 | Y | Liver, abdominal cavity | 8*9 | N |
| **3-7** | 135 | 1300 | 450 | Y | Liver, abdominal cavity | 10*11 | N |
| **3-8** | 135 | 1200 | 480 | Y | Liver, abdominal cavity | 7*8 | N |

| | | | | | | | |
|---|---|---|---|---|---|---|---|
| **3-1 (-)** | 135 | 1200 | 350 | N | - | - | Y |
| **3-2 (-)** | 135 | 1200 | 400 | N | - | - | Y |
| **3-3 (-)** | 135 | 1300 | 430 | N | - | - | Y |
| **3-4 (-)** | 135 | 1300 | 450 | N | - | - | Y |
| **3-5 (-)** | 135 | 1400 | 400 | N | - | - | N |
| **3-6 (-)** | 135 | 1100 | 350 | N | - | - | N |
| **3-7 (-)** | 135 | 1300 | 350 | N | - | - | N |
| **3-8 (-)** | 135 | 1300 | 300 | N | - | - | N |
| **4-1** | 180 | 1200 | 380 | Y | Liver, spleen, lesser curvature of stomach, pelvis | 15*12 | Y |
| **4-2** | 180 | 1100 | 420 | Y | Liver, pelvis, abdominal cavity | 33*26 | Y |
| **4-3** | 180 | 1200 | 350 | Y | Liver, spleen, abdominal cavity, pelvic cavity | 36*30 | Y |
| **4-4** | 180 | 900 | 480 | Y | Liver, abdominal cavity, pelvic cavity | 12*15 | Y |
| **4-5** | 180 | 1200 | 450 | Y | Liver, abdominal cavity, pelvic cavity | 17*20 | N |
| **4-6** | 180 | 1200 | 600 | Y | Liver, abdominal cavity, pelvic cavity | 16*13 | N |
| **4-7** | 180 | 1300 | 450 | Y | Liver, abdominal cavity | 27*22 | N |
| **4-8** | 180 | 1200 | 480 | Y | Liver, abdominal cavity | 40*34 | N |
| **4-1 (-)** | 180 | 1000 | 350 | N | - | - | Y |
| **4-2 (-)** | 180 | 1200 | 400 | N | - | - | Y |
| **4-3 (-)** | 180 | 1200 | 430 | N | - | - | Y |
| **4-4 (-)** | 180 | 1300 | 450 | N | - | - | Y |
| **4-5 (-)** | 180 | 1100 | 400 | N | - | - | N |
| **4-6 (-)** | 180 | 1300 | 350 | N | - | - | N |
| **4-7 (-)** | 180 | 1200 | 350 | N | - | - | N |
| **4-8 (-)** | 180 | 1300 | 300 | N | - | - | N |
