# Supplementary material for: Preliminary Proteomic and Metabolomic Analyses Reveal Potential Serum Biomarkers for Identifying Alveolar Echinococcosis in Mice
Source: Vet Sci. 2025 Jun 9;12(6):565. doi: 10.3390/vetsci12060565 (PMC12197404; doi:10.3390/vetsci12060565)
Supplement: Supplementary file 1 [file vetsci-12-00565-s001.zip › Materials Figure S2.pdf]

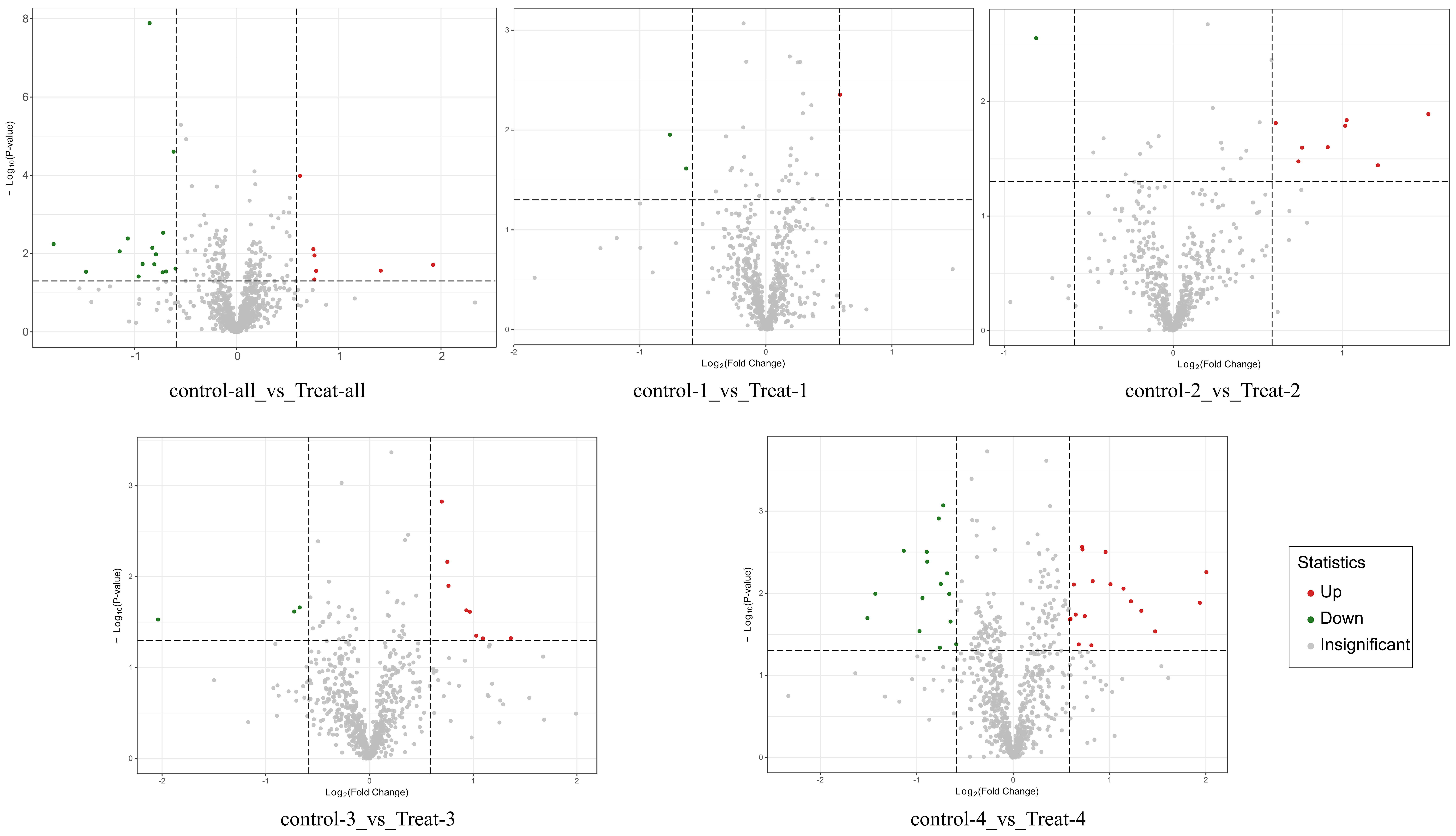

Materials Figure S2 Volcano plots of differentially expressed proteins, where the horizontal axis represents the log2 fold change, the vertical axis represents -log10 P-value, and red and green dots represent upregulated and downregulated proteins, respectively.
