# Supplementary material for: Preliminary Proteomic and Metabolomic Analyses Reveal Potential Serum Biomarkers for Identifying Alveolar Echinococcosis in Mice
Source: Vet Sci. 2025 Jun 9;12(6):565. doi: 10.3390/vetsci12060565 (PMC12197404; doi:10.3390/vetsci12060565)
Supplement: Supplementary file 1 [file vetsci-12-00565-s001.zip › Materials Figure S3.pdf]

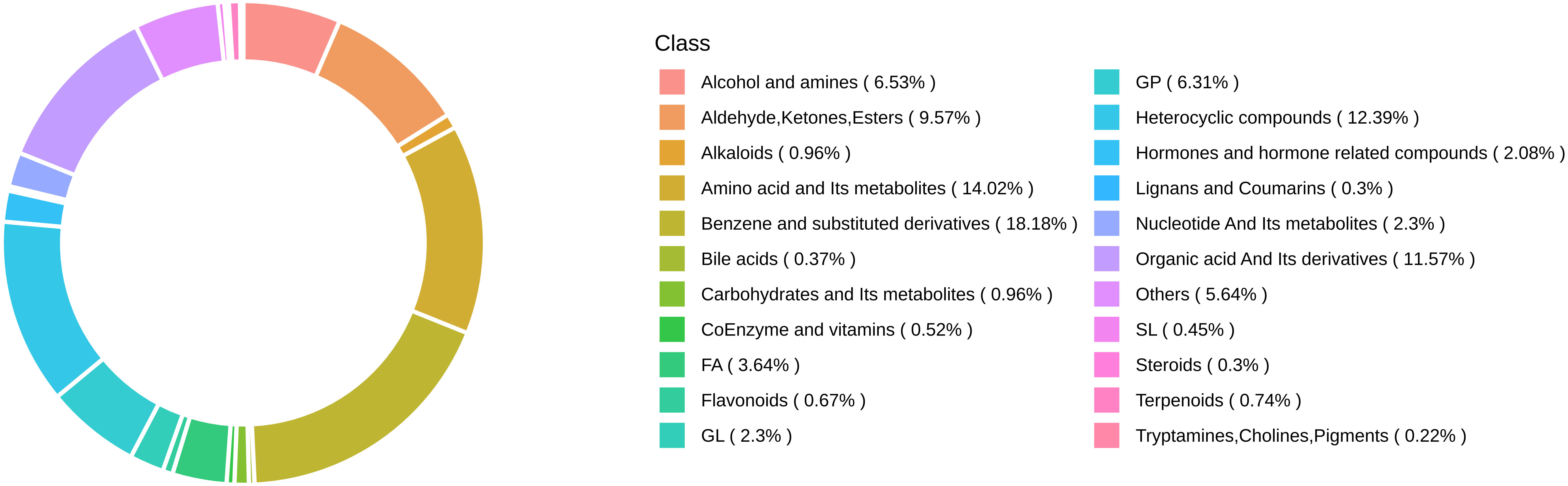

Materials Figure S3 Metabolomie supporting data for AE, mouse serum samples. Ring chart depicting the composition of metabolite categories, where eaeh color represents a different metabolile category, and the size of each colored segment represents its proportion.
